# Identifying Asbestos-Containing Materials in Homes: Design and Development of the ACM Check Mobile Phone App

**DOI:** 10.2196/formative.8370

**Published:** 2017-12-14

**Authors:** Matthew Hayden Govorko, Lin Fritschi, James White, Alison Reid

**Affiliations:** ^1^ School of Public Health Curtin University Perth Australia; ^2^ Reach Health Promotion Innovations Perth Australia

**Keywords:** application development, asbestos, asbestos-containing materials, mobile phones, smartphone, residential environment, mobile applications, environment and public health

## Abstract

**Background:**

Asbestos-containing materials (ACMs) can still be found in many homes in Australia and other countries. ACMs present a health risk when they are damaged or disturbed, such as during do-it-yourself home renovations. However, community members lack knowledge and awareness about asbestos identification and its safe management in residential settings.

**Objective:**

The objective of our study was to describe the process of developing a mobile phone app, ACM Check, that incorporates a questionnaire designed to identify and assess ACMs located in residential settings.

**Methods:**

A multidisciplinary team was involved in the formative development and creation of the mobile phone app. The formative development process comprised 6 steps: defining the scope of the app; conducting a comprehensive desktop review by searching online literature databases, as well as a wider online search for gray literature; drafting and revising the content, questionnaire, conditional branching rules, and scoring algorithms; obtaining expert input; manually pretesting the questionnaire; and formulating a final content document to be provided to the software development company. We then constructed ACM Check on the iOS platform for use in a validation study, and then updated the app, replicated it on Android, and released it to the public.

**Results:**

The ACM Check app identifies potential ACMs, prioritizes the materials based on their condition and likelihood of disturbance, and generates a summary report for each house assessed.

**Conclusions:**

ACM Check is an initiative to raise community members’ awareness of asbestos in the residential environment and also serves as a data collection tool for epidemiologic research. It can potentially be modified for implementation in other countries or used as the basis for the assessment of other occupational or environmental hazards.

## Introduction

Asbestos is the term given to a family of naturally occurring fibrous silicates that have been used in a wide variety of building materials, commonly referred to as asbestos-containing materials (ACMs) [1]. Australia was the highest per capita consumer of ACMs in the world during the mid-20th century [2]. Many of these ACMs were asbestos cement products, such as flat and corrugated asbestos cement sheeting, in which the asbestos fibers were bonded to a base material. These products were installed in residential settings between the mid-1940s and the late 1980s [3]. Until the 1960s, approximately 25% of all new Australian homes were clad with asbestos cement products [2], and it is likely that almost all houses built before 1990 contain some form of asbestos [3]. All forms of asbestos have been classified as carcinogenic [4], and a prohibition was declared on all new uses of asbestos in Australia in 2004. However, the prohibition does not extend to ACMs that were in place prior to the date the prohibition was enforced [5]. As a result, a large amount of asbestos is still present in the residential environment.

However, identifying ACMs is difficult, and householders lack knowledge and awareness regarding the identification of ACMs in and around the home and how to safely manage these materials to prevent exposure to asbestos fibers [6]. Identifying in situ ACMs is complicated by the large and varied uses of asbestos prior to its phase out. This is exacerbated by the similarities in visual features between certain older ACMs and the newer asbestos-free materials, which makes distinguishing between ACMs and non-ACMs complicated for the untrained individual. An Australian asbestos awareness survey conducted in 2014 found that participants’ confidence in their ability to identify ACMs was low, particularly among do-it-yourself (DIY) home renovators and the general public [6]. The survey established that greater practical information and guidance were needed on how to identify ACMs and how to correctly manage the risks [6].

ACMs present a health risk when they are in poor condition due to damage, deterioration, or weathering, or when they are disturbed. For instance, a significant number of asbestos fibers can be released into the air when working with asbestos cement sheeting in houses, eaves, fences, or sheds, especially when using power tools for cutting, drilling, grinding, sanding, or sawing [7,8]. This may particularly be a problem for DIY home renovators if they do not take appropriate precautions when dealing with potential ACMs.

In Australia, smartphones are owned by approximately 80% of people over the age of 18 years, with the majority of the market being held by Apple (41%) and Samsung (32%) [9]. Because asbestos identification requires close-up visual inspection of the features of various types of materials that are spread throughout different locations around the property, their high level of portability makes smartphones and tablets an ideal platform to administer an app targeting asbestos identification. Mobile apps have been developed and used to target other environmental health issues, such as air quality [10], noise monitoring [11], and sun safety and melanoma prevention [12,13]. However, no mobile apps are freely available in Australia that can be used to screen the residential property for the presence of in situ asbestos. Therefore, household occupants need to turn to an environmental consultant, asbestos inspector, industrial hygienist, or other qualified professional, which can be costly to the home owner. Similar to mobile apps that can be used as early-stage screening tools such as for prostate cancer [14] or depression [15], a mobile app that screens for asbestos can be a tool that empowers users to approach the issue and take the first step toward prevention, action, or remediation.

The aim of this paper is to describe the design and development of the mobile app ACM Check (short for Asbestos-Containing Material Check). ACM Check is an initiative to raise community members’ awareness of asbestos in the home environment and also serves as a data collection tool for epidemiologic research.

## Methods

The development of ACM Check was approved by the Human Research Ethics Committee (RDHS-89-15) of Curtin University, Perth, Australia.

### The Multidisciplinary Research and Development Team

We developed ACM Check in a collaborative partnership involving occupational epidemiologists and a doctoral student in public health and epidemiology from the School of Public Health, Curtin University; scientific health officers and toxicologists from a state environmental health agency (Environmental Health Directorate, Western Australia Department of Health, Perth); and a health promotion software development company (Reach HPI, Perth). Following advice from previous health promotion-based and researcher-led app development projects [16-18], we involved the software developer early on in the process due to the need for specialized development skills when developing native mobile apps (versus other communication technologies, such as text messaging or websites). We engaged the software development company to provide guidance surrounding the technical aspects of mobile app development and to bring expertise in the field of graphic design, user interface design, and user experience design.

### Development Process

The development of the app was an iterative process that occurred in 2 broad phases: formative development, followed by creation of the mobile app. The formative development process comprised the following steps: planning and defining the scope of the app; conducting a comprehensive desktop review; drafting and revising the content, questionnaire, conditional branching rules, and scoring algorithms; obtaining expert consultation and input; manually pretesting the questionnaire; and formulating a final content document, which was provided to the software development company.

#### Phase 1: Formative Development

In the first stage of the formative development process, we defined the scope and aim of the app. We clarified the target end users, the rationale for development, the functions we wanted to include, the data outputs we wanted to generate, and how these aims would be achieved (Table 1).

We undertook a comprehensive desktop review of scientific peer-reviewed journal articles obtained from online databases, including PubMed, ProQuest, and ScienceDirect, and gray literature obtained from Australian government and nongovernment websites, such as the Asbestos Safety and Eradication Agency and state health department websites, prior to drafting the content for the app. We also reviewed the reference lists of the publications for additional relevant literature. Search terms were “asbestos” OR “asbestos-containing materials” AND “identification,” “survey,” “questionnaire,” “assessment,” “material assessment,” “exposure assessment,” “risk assessment,” and “condition assessment.” Documents published by the Australian federal and state government health authorities were the primary basis of the background information used in the app.

**Table 1:** Scope of ACM Check.

Key Factor	Parameters of ACM Check
Problem	Difficulties visually identifying ACMs^a^ in residential settings
	Lack of awareness among DIY^b^ renovators
Target audience	Householders, particularly DIY renovators
	Local government environmental health officers
	Tradespeople working in the residential sector
Setting	Residential settings in Western Australia, which excludes commercial, industrial, and waste sites
Objectives	Identify in situ ACMs inside and outside homes
	Assess current condition and likelihood of disturbing the ACMs
	Direct users to further resources that assist in the safe management of asbestos
	Collect questionnaire data regarding the amount, type, and condition of ACMs
Method	Conduct a self-administered questionnaire using automated conditional branching (if-then rules) and an additive scoring algorithm for priority assessment
	Generate a summary report for each completed home inspection
	Provide links to relevant information, resources, and contacts
Significance	Increase users’ awareness of asbestos in the residential environment
	Inform relevant government and nongovernment agencies about the current amount and condition of ACMs in Western Australian households

^a^ACM: asbestos-containing material.

^b^DIY: do-it-yourself.

We also searched for examples of different ways to assess the condition and exposure potential of ACMs in residential or occupational settings. We held meetings with the development team to help define the scope of the app and determine what areas or materials are likely to be of most significance in the community. We sought input from 9 further experts and stakeholders outside of the development team after we had made some revisions of the content and questionnaire, including local government environmental health officers, environmental consultants, and asbestos removalists.

We pretested the questionnaire using pen and paper to test the practicality of the questions and instructions, to test the flow of the conditional branching (if-then rules), and to assess the scoring algorithms. The outputs, such as probabilities of each key material containing asbestos and its overall rating, were calculated manually at the completion of each trial. The pretesting also provided approximations for the time it would take to complete the inspection and questionnaire once it was in the digital format. We revised and finalized the questions, conditional branching, scoring algorithms, and content of ACM Check based on these manual trials and expert reviews.

#### Phase 2: Creation of the Mobile App

We provided the final questionnaire and content to the software development company. ACM Check was initially developed for the iOS platform (Apple Inc). Developing the app first for one platform, then refining it before building the app for other platforms, is an efficient approach that minimizes the cost of iterating multiple versions [18]. We chose the iOS platform for the initial version due to the smaller number of devices for testing, and the fact that Apple had the largest market share in Australia at the time of initial development [9]. After the initial build, we used TestFlight (Apple Inc) for iOS to beta test and debug ACM Check.

We then trialed the iOS version of ACM Check on a sample of metropolitan homes in Perth, Western Australia. We obtained user feedback to further improve the accuracy, functionality, and usefulness of the app before releasing it to the public. The iOS version of ACM Check was modified based on user feedback before being replicated and developed for Android (Google Inc). We released ACM Check to the public via the App Store (Apple Inc) and Google Play (Google Inc) in June 2017.

## Results

The app delivers a self-administered, structured questionnaire that is supplemented with easy-to-follow instructions and images of ACMs. There are 3 modules that make up the questionnaire. The first module collects data on user and housing information, including state of residence, user description (eg, community member, householder, or DIY renovator; local government environmental health officer; or tradesperson working in residential settings), residential post code, period of house construction, type of dwelling, and number and age category of occupants. The second and third modules aim to identify potential ACMs located outside and inside the home, respectively. To do this, the questionnaire methodically guides the user through a visual inspection of locations around the house where key materials that may contain asbestos could be located. The outside locations inspected include the exterior walls and gable ends, eaves or soffit linings, roofing, gutters, downpipes, electrical meter box, fencing, and outbuildings. The inside locations inspected include the interior walls, cupboards and backsplashes, ceilings, flooring, and heater flues.

### Questionnaire Design

The ACM Check questionnaire is a computerized, self-administered questionnaire that uses conditional branching (“skip logic”) to assign each screened material a probability of containing asbestos, and subsequently to assign each potential ACM a priority level for action or remediation. The answers of the completed sections and modules are linked using if-then rules. For example, *if* the house was built before 1985 *then* it is highly likely to have ACM present. This feature results in a custom pathway being created through the questionnaire. Consequently, users are automatically navigated through the questionnaire in an efficient manner so that they do not need to read and answer all of the questions [19].

### Screening for Asbestos-Containing Materials

**Figure 1:**
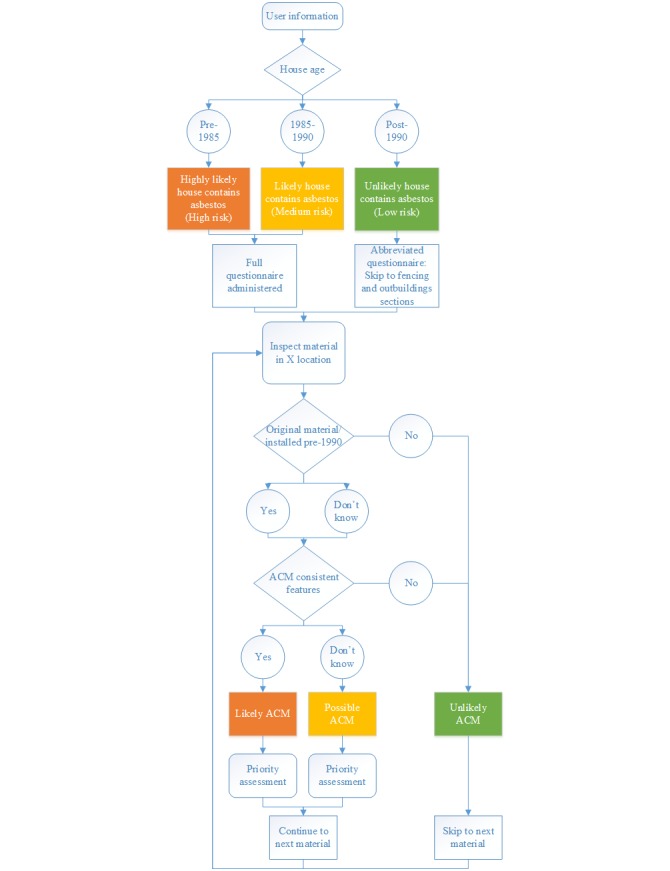
Process flow chart showing the key factors used in the ACM Check app to determine the probability that asbestos is present in a material or location. ACM: asbestos-containing material.

The app uses multiple-choice questions to assess each location inside or outside the house (Figure 1). The information necessary for the visual identification of ACMs includes (1) the age of the house, (2) its renovation history, (3) the location or use of the ACM, and (4) visual features specific to each type of material.

The age of the house is relevant because, in Australia, asbestos was phased out of residential building products that were manufactured in the years leading up to 1987 [20]. However, builders or tradespeople may have had stockpiles of ACMs in their warehouses or trade centers that were used beyond that date. Therefore, we used a conservative cutoff date of 1990 in the app. More specifically, we used 3 categories for the probability that a house contains asbestos based on the age of the house to best reflect the years in which ACM use was phased out of residential buildings (Figure 1). We adapted these categories from rankings used by the Environmental Health Standing Committee [3]. The answer to this question also determines whether the full questionnaire or only sections of it will be administered to the user. If the house was built after 1990 (the date ACMs ceased to be installed in new housing), then an abbreviated questionnaire is administered that only asks questions relating to outside materials, such as fences or outbuildings, that could be present from earlier developments (Figure 1).

For pre-1990 homes, ACMs may have been replaced with non-ACMs. Therefore, each material screened in the app has a question relating to date of installation or its renovation history.

The final factor in screening for the likelihood of a material containing asbestos is to inspect the visual features. Although some ACMs appear visually identical to non-ACMs, other materials can have distinct visual features that indicate whether they are likely to contain asbestos.

Based on the user’s answers to questions regarding these 4 factors, each material or location inspected is automatically designated as 1 of 4 probabilities of containing asbestos: not applicable, unlikely ACM, possible ACM, or likely ACM. The designation of not applicable is used only for those materials or locations that are not present inside or outside of the home as indicated by the user. For example, not all properties have an outbuilding or a permanent internal heater, so when these are not present they are designated not applicable. The designation of possible ACM is used to highlight the situations where it is more difficult to confirm or rule out the probability that a material contains asbestos. This can be due to difficulties in visual identification, such as a lack of visual characteristics that distinguish ACM from non-ACM, or lack of information on the year of installation or the renovation history. For instance, if a user indicates they have eaves made of cement sheeting with joiner strips, but they do not know if they were installed before 1990 or replaced after 1990, then those eaves are designated as possible ACM.

### Priority Assessment of Possible and Likely Asbestos-Containing Material

The mere presence of in situ ACMs in or around the home does not necessarily mean individuals are inhaling or being exposed to asbestos fibers, or that they are at an increased risk of developing an asbestos-related disease. Two key variables that need to be considered when looking at the risk of asbestos exposure in the residential environment are the current condition of the ACM and the likelihood of disturbing the ACM. For instance, an asbestos cement product that is in good condition and left undisturbed is associated with a minimal risk of asbestos exposure and presents a negligible health risk [5]. In contrast, an asbestos cement product in poor condition or that is accidentally or deliberately disturbed can result in dispersal of asbestos fibers into the air and is associated with a greater risk of exposure [5]. Therefore, a priority assessment that incorporates these 2 factors is triggered for each material that is designated a probability of possible or likely ACM.

The current condition is based on the degree to which the ACM shows signs of weathering, deterioration, or physical damage, such as surface marks, scratches, cracks, splits, breakages, or water damage, and on how friable it is; that is, how easily the material crumbles. There are 2 questions pertaining to the material’s condition: a qualitative and a quantitative assessment. The qualitative question has 4 possible outcomes: “good,” “fair,” “poor,” and “very poor.” Descriptive text accompanies each option to help the user select the most appropriate answer. Additionally, the user is asked a quantitative question, which has the user rate the material on a scale of 1 (very poor) to 10 (very good).

The likelihood of disturbance refers to the probability of the ACM being damaged or disturbed in the near future. This reflects the chances of asbestos fibers being released from the material and made airborne, which subsequently increases the risk of their inhalation by occupants in their vicinity. ACMs can be disturbed for a variety of reasons, including through access, use, repair, or renovation and maintenance activities. The likelihood of disturbance is also presented as a multiple-choice question with the user having to select 1 of 4 answers: “unlikely,” “somewhat likely,” “likely,” or “highly likely,” which are accompanied by descriptive text.

**Figure 2:**
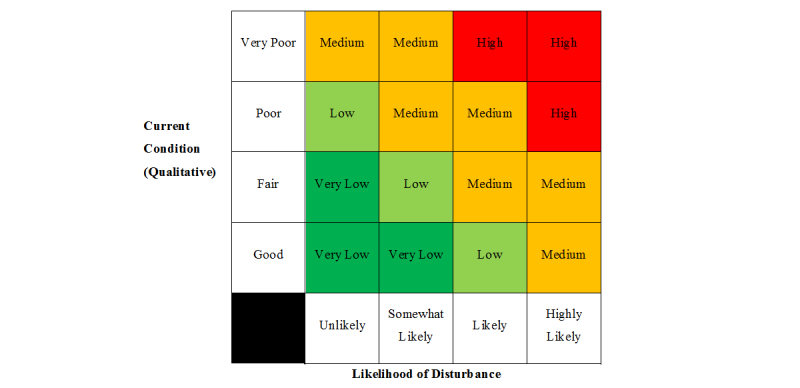
Risk matrix used to give a priority level for action or remediation to each possible or likely asbestos-containing material.

The answers to the questions on qualitative condition and likelihood of disturbance are assigned numerical values, which are then summed to provide an overall rating to the ACM, expressed as a priority level (Figure 2). The priority level, either “very low,” “low,” “medium,” or “high” priority, indicates which ACMs are of most concern to that property with respect to the potential risk of asbestos exposure and which ACMs require remediation. For example, an ACM assigned as high priority should be given greater attention by the user and has a greater risk of releasing asbestos fibers than an ACM that is given a very low priority.

### Summary Report

A summary report is generated after the inspection has been completed, which shows the user the probability of each material assessed containing asbestos, its current condition, the likelihood of disturbance, and the priority level for each possible or likely ACM (see Multimedia Appendix 1). Depending on the priority level, a general recommendation is provided for each ACM. These range in severity, from “Monitor and no immediate action necessary,” “Monitor and minor maintenance and repair,” and “Removal and replacement should be a priority. Major repair activity should be considered as a secondary and temporary action,” through to “Consult an asbestos professional for removal, disposal and replacement of the ACM.” These recommendations are presented in table format alongside the corresponding and color-coded priority level. Furthermore, each recommendation is accompanied by a description and links to further relevant resources where possible.

All summary reports are stored in the ACM Check Reports tab on the home screen for quick reference (see Multimedia Appendix 2). This allows users to complete the app on multiple homes, which is useful for owners of multiple properties or individuals who work in multiple residential settings.

## Discussion

ACM Check is a screening tool designed to identify and assess the condition of potential ACMs in situ in residential settings. The app directs users to further information from reputable authorities pertaining to asbestos and its safe management. ACM Check can also be used as a data collection tool for researchers working with relevant government and nongovernment agencies to map the presence and condition of ACMs in the built environment. Furthermore, ACM Check is freely available to use to assist with asbestos identification and to raise awareness about the hazards of asbestos exposure.

In situ asbestos is an ongoing problem in Australia despite being phased out of residential building products during the 1980s. ACM Check offers a free, quick, and easy-to-follow solution that will aid in the prevention of exposure to in situ asbestos in the residential environment. ACM Check is, to our knowledge, the first and only mobile app available on the market that guides users through a visual inspection of the home from beginning to end in a systematic manner. To motivate people to use the app, we promoted ACM Check via live interviews on community radio stations, as well as through social media and Web posts by various not-for-profit organizations that target asbestos-related disease prevention and awareness. Furthermore, ACM Check was promoted on trade union and occupational health and safety-related websites to encourage workers to download and use the app.

The app could be adapted for use in other countries where ACMs were used in residential settings. The questions and rules are likely to need careful modification if this tool is to be adopted for use in another country with a history of asbestos use that is different from that in Australia. For instance, different countries may have phased out asbestos in different years (if applicable); have different regulations and prohibitions pertaining to asbestos use; and have different profiles, types, and frequencies of ACMs used in homes. Regardless, ACM Check offers a model that could be easily modified to accommodate country-specific variables. Similarly, ACM Check could be expanded or modified to target asbestos in occupational settings, or used as a roadmap for new apps targeting the identification of other occupational hazards.

### Limitations

ACM Check does not replace or eliminate the need for consultation with an asbestos professional. ACM Check attempts to capture the main sources and locations where ACMs are likely to be present in residential settings. However, it is impossible to capture all scenarios and materials that could contain asbestos in the residential environment due to the large and diverse uses of asbestos in the past [1].

### Conclusion

ACMs are difficult for the untrained eye to identify in the built environment, but to prevent exposure to asbestos, identification is necessary. As a multidisciplinary team, we designed and developed a practical and easy-to-use mobile app, ACM Check, to screen for in situ ACMs in the residential environment. ACM Check forms part of a primary prevention strategy aimed at minimizing users’ risk of exposure to asbestos fibers in the residential environment while doubling as a scientific data collection tool. This technology could be modified to raise awareness among the broader community about other environmental health issues.

## Appendix

Multimedia Appendix 1 Sample report generated by ACM Check showing a summary of a completed inspection and the table of general recommendations.

Multimedia Appendix 2 Screenshots showing the home screen and sections of the questionnaire from the ACM Check app.

## Abbreviations

ACM: asbestos-containing material
DIY: do-it-yourself
