# Supplementary material for: Identifying Asbestos-Containing Materials in Homes: Design and Development of the ACM Check Mobile Phone App
Source: JMIR Form Res. 2017 Dec 14;1(1):e7. doi: 10.2196/formative.8370 (PMC6334671; doi:10.2196/formative.8370)
Supplement: Multimedia Appendix 1 [file formative_v1i1e7_app1.pdf]

ACM Report, 26 June 2017

Postcode: 6148	House built: Pre 1985			
Probability house contains asbestos based off house age: Likely				
**Product type**	**Probability of being an ACM**	**Current condition**	**Potential for disturbance**	**Overall rating**
**Outside**				
Exterior wall cladding	Unlikely			
Eaves / soffit lining	Likely	Good	Unlikely	Very low priority
Roofing	Unlikely			
Gutters	Unlikely			
Drainpipes / downpipes	Unlikely			
Backing board in electric meter box	Likely	Good	Somewhat likely	Very low priority
Fencing	Likely	Fair	Unlikely	Very low priority
Outbuilding roof	Unlikely			
Outbuilding walls	Unlikely			
**Inside**				
Interior walls	Unlikely			
Backing to wall tiles	Possible	Good	Unlikely	Very low priority
Ceiling	Unlikely			
Linoleum or vinyl tile flooring	Likely	Good	Unlikely	Very low priority
Heater flue pipe	N/A			

Recommendations

- If you wish for the possible or likely ACM identified in this app to be confirmed as containing asbestos, we recommend that you have the suspect ACM sampled.
- However, members of the public are advised not to undertake destructive sampling (i.e. sampling that involves cutting or breaking the material) and/or any sampling which involves friable ACMs, such as asbestos pipe lagging or asbestos fuses. To have these materials sampled please contact a qualified asbestos professional and have it tested at a laboratory accredited by NATA for asbestos analysis.
- A list of certified labs can be found here or by calling 08 9486 2800.
- To conduct passive sampling, i.e., collecting tape dust samples or picking up suspect ACM fragments, please refer to section 6.1 'Sampling and Analysis' found in the following document: Guidance Note: Identification of asbestos containing material.
- Based on the overall rating of each possible or likely ACM (i.e. very Low priority, low priority, moderate priority, high priority), consult the following table for general recommendations.

**OVERALL RATING**	**GENERAL RECOMMENDATIONS / ACTIONS**
Very low priority	**Monitor and no immediate action necessary.**<br>Monitoring of potential ACM for any signs of visible deterioration or damage. ACM in good condition may benefit from preventive maintenance actions including painting, sealing or encapsulating.
Low priority	**Monitor and minor maintenance/repair.**<br>Monitoring of the ACM should be conducted frequently. Maintenance and repair procedures should be considered as a short term measure for any minor damages (sealing cracks and surface scratches, painting the product if appropriate etc.) with plans for removal in the long term (pending on monitoring of the condition of the ACM).
Moderate priority	**Removal and replacement should be a priority. Major repair activity should be considered as a secondary and temporary action.**<br>Maintenance and repair may be of insufficient benefit. It is recommended that you have the ACM removed and replaced. It is suggested that you contact your local government environmental health officer for advice and consider using the services of a licensed asbestos professional (depending on the work to be undertaken, this may be either a restricted or unrestricted asbestos removal license holder).
High priority	**Consult an asbestos professional for removal, disposal and replacement of the ACM.**<br>Removal and replacement of the product as soon as possible is strongly recommended. It is highly recommended that you consult with an asbestos professional (e.g., local government environmental health officer, occupational hygienist, licensed asbestos removalist) for advice on how to deal with the ACM. For certain ACMs (i.e., friable ACMs) that are to be removed, it is strongly recommended that a professional with an Unrestricted Asbestos Removal License obtained from WorkSafe be used to remove and dispose of the material. View a list of Unrestricted Asbestos Removal License holders based in WA.
