# Supplementary figures and images for: Identifying Asbestos-Containing Materials in Homes: Design and Development of the ACM Check Mobile Phone App
Source: JMIR Form Res. 2017 Dec 14;1(1):e7. doi: 10.2196/formative.8370 (PMC6334671; doi:10.2196/formative.8370)

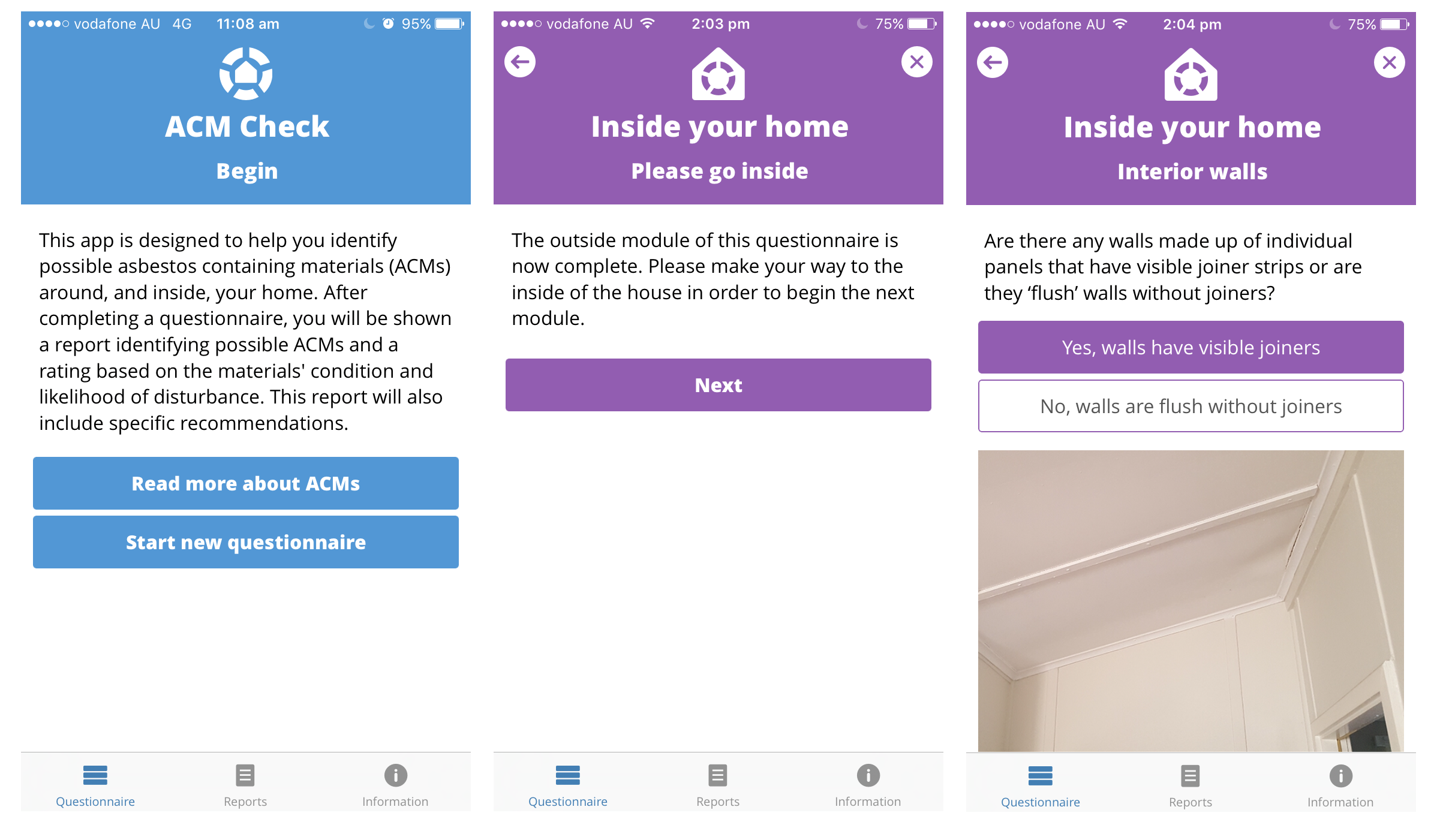

Supplement: Multimedia Appendix 2 [file formative_v1i1e7_app2.png]
